# N-Doped Biochar as a New Metal-Free Activator of Peroxymonosulfate for Singlet Oxygen-Dominated Catalytic Degradation of Acid Orange 7

**DOI:** 10.3390/nano11092288

**Published:** 2021-09-02

**Authors:** Ruirui Han, Yingsen Fang, Ping Sun, Kai Xie, Zhicai Zhai, Hongxia Liu, Hui Liu

**Affiliations:** 1 College of Advanced Materials and Engineering, Jiaxing Nanhu University, Jiaxing 314001, China; hanruirui@zjxu.edu.cn (R.H.); liuhongxia@zjxu.edu.cn (H.L.); 2 College of Biological, Chemical Sciences and Engineering, Jiaxing University, Jiaxing 314001, China; fangyingsen@zjxu.edu.cn (Y.F.); sunping@zjxu.edu.cn (P.S.); xiekai2050956@163.com (K.X.); zhaizhicai@zjxu.edu.cn (Z.Z.); 3 College of Petroleum Engineering, Liaoning Petrochemical University, Fushun 113001, China

**Keywords:** nitrogen-doped biochar, permonosulfate (PMS), singlet oxygen, acid orange 7

## Abstract

In this paper, using rice straw as a raw material and urea as a nitrogen precursor, a composite catalyst (a nitrogen-doped rice straw biochar at the pyrolysis temperature of 800 °C, recorded as NRSBC800) was synthesized by one-step pyrolysis. NRSBC800 was then characterized using XPS, BET, TEM and other technologies, and its catalytic performance as an activator for permonosulfate (PMS) to degrade acid orange 7 (AO7) was studied. The results show that the introduction of N-doping significantly improved the catalytic performance of NRSBC800. The NRSBC800/PMS oxidation system could fully degrade AO7 within 30 min, with the reaction rate constant (2.1 × 10 ^−1^ min^−1^) being 38 times that of RSBC800 (5.5 × 10^−3^ min^−1^). Moreover, NRSBC800 not only had better catalytic performance than traditional metal oxides (Co_3_O_4_ and Fe_3_O_4_) and carbon nanomaterial (CNT) but also received less impact from environmental water factors (such as anions and humic acids) during the catalytic degradation process. In addition, a quenching test and electron paramagnetic resonance (EPR) research both indicated that AO7 degradation relied mainly on non-free radical oxidation (primarily singlet oxygen (^1^O_2_)). A recycling experiment further demonstrated NRSBC800’s high stability after recycling three times.

## 1. Introduction

With the rapid development of the industrial economy in recent years, the number of organic pollutants discharged from industries, such as chemical plants, paper making, textile printing and dyeing, and pharmaceutical and other industries, have gradually increased. They pose a serious threat to the survival and health of humans and other organisms [1,2,3,4]. As a result, the treatment of organics in water has garnered increased attention. At present, the main treatment methods for organics in wastewater include physical [5,6], chemical and biological methods [7,8,9,10,11,12], or a combination thereof [13,14,15]. Among others, advanced oxidation methods showed potential advantages in treating organics, including the generation of reactive free radicals with strong oxidizing abilities, an effective degradation of organics and a better mineralizing effect [16]. Peroxymonosulfate (PMS)-based technology is a recently emerging type of advanced oxidation technology. It effectively degrades organic pollutants by generating strongly oxidizing sulfate radicals. Common activation methods of PMS include photo and thermal methods [17,18,19], microwave, transition metal ions, zero-valent iron, etc. [20,21,22,23,24,25,26,27,28,29,30]. However, these methods have been found to have defects such as loss of metal ions, high energy consumption, etc. Therefore, the development of new catalysts with low or even zero metal loss and no consumption of other energy has become a significant focus for current research.

In recent years, metal-free catalysts have aroused wide concern [31,32]. Carbon nanomaterials, such as carbon nanotubes (CNTs) and graphene, have surface chemical inertness, good electric conductivity, high specific surface area, large pore volume, and have been proven to be of good catalytic effect in different kinds of degradation [33,34]. Therefore, the introduction of carbon nanomaterials into environmental catalysis as metal-free heterogeneous catalysts for the removal of organic pollutants in water is very promising [35,36,37,38,39]. The doping of heteroatoms including nitrogen, sulfur, phosphorus and boron in carbon nanomaterials is an effective method for achieving higher catalytic activity from such materials and has become a research focus in recent years [40,41,42,43].

Moreover, there are two major PMS activation mechanisms currently, namely the free radical mechanism and the non-free radical mechanism. Over time, the former mechanism has been taken up as the main method of PMS oxidation and removal of pollutants. Recently, however, scientific researchers have noticed that the non-free radical oxidation pathway has an advantage in many aspects when compared with the free radical oxidation pathway [44,45,46]. Singlet oxygen is a selective oxidant of higher activity which receives less water interference and is relatively stable and more effective in degrading organic micro-pollutants in the environment in comparison with hydroxyl radicals and sulfate radicals [47]. In light of the different oxidative mechanisms in PMS activation, the development of efficient, stable and economical PMS activators and PMS activation mechanisms has attracted a great deal of attention from many researchers [48,49,50].

However, at present, the manufacturing cost of carbon nanomaterials is high, which limits their practical application in wastewater treatment. Thanks to the excellent performance of carbon nanomaterials in activating PMS, biomass-derived carbon materials (biochars, BCs) have gradually attracted more and more attention in recent years. Biomass is waste and garbage in daily life. It is cheap and easy to obtain. Therefore, BCs have become increasingly important as a solution to remediate pollutants in the environment [51,52,53]. Although, as green adsorbents, BCs have been widely used in soil improvement and wastewater treatment, as PMS activators, their mechanism of activation needs further systematic research [54,55,56,57,58,59,60].

In this research, using rice straw as a raw material, nitrogen-doped biochar was prepared under different pyrolysis temperatures with urea as the nitrogen source. The resulting materials then went through SEM, TEM, XRD, BET, XPS, EPR and Raman characterization. The removal effect of the biochar/PMS system on AO7 in water was also studied. Moreover, the effect of reaction conditions on the catalytic performance was analyzed, and the repeated use of the catalyst in the reaction system and its application in actual water was explored. It is expected to provide a scientific basis and technical support for the application of straw biochar in PMS activation.

## 2. Materials and Methods

### 2.1. Reagents and Materials

The hydrophilic PTFE syringe filter (PTFE, 0.45 µm) was purchased from ANPEL lab. Tech. Inc. (Shanghai, China). AO7 (C_16_H_11_N_2_NaO_4_S, AO7, ≥98%), potassium monopersulfate (PMS, ≥47%), p-benzoquinone (p-BQ, ≥99%), tertiary butyl alcohol (TBA, ≥99.5%), l-histidine (L-His, ≥99%), ethanol (EtOH, ≥99.7%) and methanol were of chromatographic grade, and all other materials were analytically pure and purchased from Shanghai Macklin Biochemical Co., Ltd. (Shanghai, China). The rice straw (RS) was from rural areas around Jiaxing, washed in deionized water, dried, comminuted by a crusher and sifted through a 100 mesh sieve. The lab water was ultrapure water.

### 2.2. Experimental Methods

#### 2.2.1. Catalyst Preparation

Nitrogen-doped rice straw biochar (NRSBC) was prepared by pyrolysis. The specific steps were as follows: wash, clean and dry the straw; comminute it into power with a high-speed crusher; sieve (100 mesh) and weigh a certain amount of the rice straw powder and the ground urea; mix them to a specific ratio and add the mixture into the quartz boat; place it in the tubular furnace; heat to the appropriate temperature at a rate of 10 °C/min in a nitrogen atmosphere (calcination temperature: 700 °C, 800 °C and 900 °C) for 2 h; cool it down; and grind it to obtain the nitrogen-doped rice straw biochar, recorded as NRSBC.

#### 2.2.2. Characterization Methods

The carbon content and the distribution of nitrogen in the catalyst were measured with an ESCALAB 250XI X-ray photoelectron spectroscopy (XPS) system (Thermo Fisher Scientific, Waltham, MA, USA); the crystal morphology of the catalyst was observed with a JEM-2100F transmission electron microscope (TEM) (JEOL, Tokyo, Japan) and a Quanta400FEG scanning electron microscope (SEM) (FEI, Hillsboro, OR, USA); the crystal structure of the catalyst was characterized by the Shimadzu XR-7000 diffractometer (XRD, Tokyo, Japan); and the specific surface area and pore size distribution of the catalyst were measured with a TriStar II 3020 specific surface area and porosity analyzer. Raman spectroscopy was collected using a Raman microscope (LabRAM HR Evolution, HORIBA JY, Paris, France).

#### 2.2.3. Catalytic Degradation Experiment

The experimental procedures were as follows: add 100 mL ultrapure water and the prepared 50 mg/L AO7 solution to the conical flask; then, add a certain amount of PMS to the reaction solution. Oscillate at 150 r/min in a water bath thermostatic oscillator at 25 °C; then, add the catalyst NRSBC and start timing. Collect a 1.0 mL sample with the pipette at 0 min, 5 min, 10 min, 15 min, 20 min, 30 min and 45 min; then, filter with a 0.45 µm syringe filter and inject into a sampling tube containing 1.0 mL methanol as a quencher. The absorbance of the solution was measured with a MAPADA UV-1100 spectrophotometer at 484 nm.

#### 2.2.4. Analytical Methods

The remaining concentration of pollutants in the samples was measured with a liquid chromatograph LC-20A (Shimadzu, Tokyo, Japan). The chromatographic column was Zorbax SB-C18 (4.6 × 250 mm, 5 μm) (Agilent, Santa Clara, CA, USA). The mobile phase was the mixture of methanol (A) and 0.3% formic acid solution (B), with the flow rate being 1.0 mL/min, and the column temperature being 30 °C. The detector was a photodiode array detector (SPDM20A).

For electron paramagnetic resonance (EPR) analysis, the EPR analyzer (Bruker A320, Karlsruhe, Germany) was employed to detect reactive oxygen species (ROS) generated in the system. The probes used were 5,5-dimethyl-1-pyrroline (DMPO) and 2,2,6,6-tetramethy l-4-piperidinone (TEMP).

## 3. Results and Discussion

### 3.1. Catalyst Characterization

**Figure 1:**
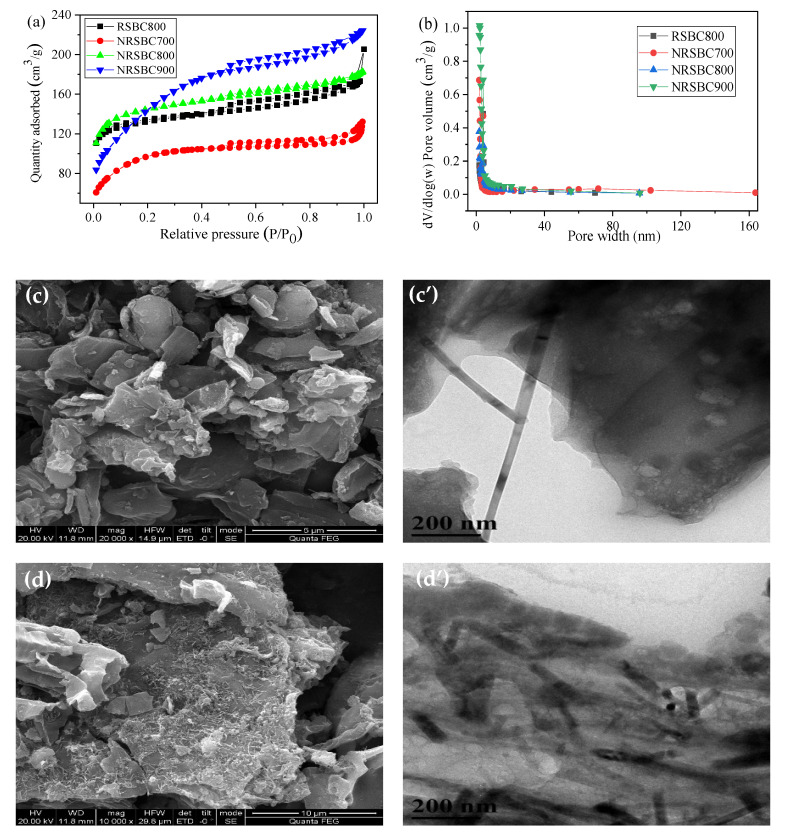
N_2_ sorption isotherms (**a**) and pore size distributions (**b**) of different materials (RSBC800, NRSBC700, NRSBC800 and NRSBC900); SEM and TEM images of RSBC800 (**c**,**c’**) and NRSBC800 (**d**,**d’**).

Figure 1a,b and Table S1 show the results of specific surface areas and pore sizes of biochar (RSBC800) prepared at the pyrolysis temperature of 800 °C, and nitrogen-doped biochar (NRSBC700, NRSBC800 and NRSBC900) prepared at different temperatures (700 °C, 800 °C and 900 °C). According to the above results and the SEM/TEM images (Figure 1c,c’,d,d’), the specific surface area of the NRSBC800 was 471.12 m^2^/g, and the pore size was mostly under 5 nm, indicating a large number of mesopores and micropores and a small number of macropores. Compared with RSBC800, biochar without nitrogen doping was smooth on the surface, mainly laminated in structure, and had a specific micron pore-like structure. During preparation of the nitrogen-doped biochar, along with gasified effusion of the pyrolysis products, the arrangement of the carbonized layers was gradually structured and the solid products showed a dispersive pore structure, making it a good carrier of adsorbents, activators and catalysts. The specific surface areas of NRSBC700, NRSBC800 and NRSBC900 were 333.7 m^2^/g, 471.1 m^2^/g and 514.3 m^2^/g, respectively; the micropore volumes were 0.2 cm^3^/g, 0.1 cm^3^/g and 0.4 cm^3^/g, respectively; and the pore sizes were 2.9 nm, 3.3 nm and 2.9 nm, respectively. Therefore, as the pyrolysis temperature increased, the specific surface area of the nitrogen-doped biochar also increased. The overall micropore volume increased slightly, but there was no significant change in pore size.

**Figure 2:**
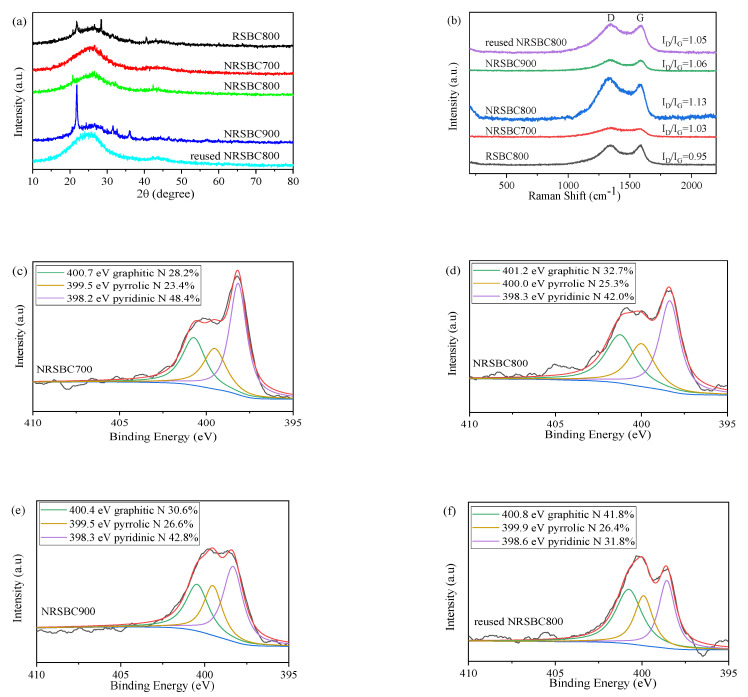
XRD patterns (**a**) and Raman spectra (**b**) of different materials (RSBC800, NRSBC700, NRSBC800, NRSBC900 and reused NRSBC800); N1s spectrum of NRSBC700 (**c**), NRSBC800 (**d**), NRSBC900 (**e**) and reused NRSBC800 (**f**).

As shown in Figure 2a, RSBC800 showed a gentle diffusion–diffraction peak at 2θ 20−35°, with many sharp small diffraction peaks. NRSBC700, NRSBC800 and NRSBC900 showed changes in the XRD spectra, of which the spectra of NRSBC700 and NRSBC800 were closer, with an enhanced gentle diffusion peak at 2θ 20−35°, indicating contracted interlamellar space, increased stacking density and higher crystallinity of cellulose graphite crystallites in nitrogen-doped biochar. NRSBC700 showed no sharp small diffraction peaks, while NRSBC800 still had some before and after the gentle diffusion peak. Compared with NRSBC700 and NRSBC800, the gentle diffusion peak of NRSBC900 at 20−35° was weaker, but NRSBC900 had a stronger sharp diffraction peak at 21° and many sharp small diffraction peaks in 22−40°, which were related to the mineral salt in the biochar. Therefore, NRSBC800 contained a lot of graphite-like microcrystalline cellulose carbon.

According to the Raman test curve in Figure 2b, peaks at 1364 cm^−1^ and 1580 cm^−1^ correspond to two characteristic absorption peaks of graphite, i.e., the D-peak and G-peak. D-to-G peak intensity ratio I_D_/I_G_ can reflect the degree of graphitization and the integrity of the carbon material. The biochar samples’ I_D_/I_G_ ≈ 1.1, indicating smaller microlites on the surface, more unsaturated carbon atoms on the surface and at the edge, a lower degree of graphitization and no fixed carbon structure. Therefore, the surface reactivity of the carbon material was relatively high, making it suitable for the activation of PMS.

Table S2 and Figure S1 show the elements and chemical status of different carbon materials. According to the XPS full-spectrum (Figure S1a), the XPS curves of RSBC800 as well as NRSBC700, NRSBC800 and NRSBC900 showed three characteristic peaks, corresponding to C 1s (285.08 eV), N 1s (399.08 eV) and O 1s (531.08 eV). The nitrogen contents of NRSBC700, NRSBC800 and NRSBC900 were 18.35%, 4.87% and 0.12%, respectively, indicating the drop in nitrogen content in the materials along with the increase in calcination temperature. The carbon contents of NRSBC700, NRSBC800 and NRSBC900 were 67.0%, 78.7% and 72.7%, respectively, meaning that biochar prepared at higher temperature was of higher carbonization degree. As shown in Figure S1b, the N/C of NRSBC900 was the smallest, that of NRSBC700 was the biggest and that of NRSBC800 was moderate, which might lead to differences in subsequent performances for PMS activation. According to the high-resolution XPS spectrogram of N 1s (Figure 2c–f), N existed in three forms in the nitrogen-doped RSBC, namely pyridine N, pyrrole N and graphite N, with slightly different relative contents at different calcination temperatures. In NRSBC800, the contents of pyridine N, pyrrole N and graphite N were 32.7%, 42.0% and 25.3%, respectively, with the content of graphite N being higher than that in NRSBC700 and NRSBC900. In general, graphite N is one of the most active sites in oxygen reduction reaction and other catalytic reactions; a higher graphite N content might enhance catalytic activity.

### 3.2. Influencing Factors of AO7 Degradation

**Figure 3:**
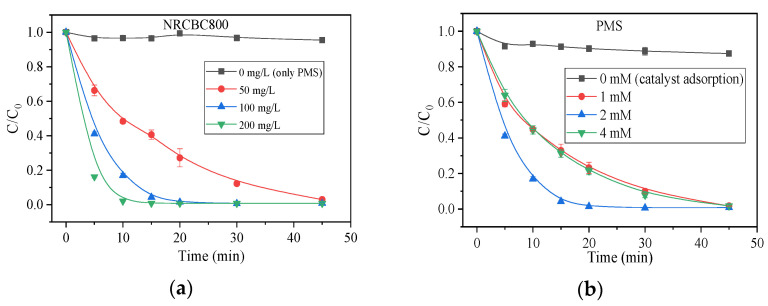
Influences of NRSBC800 dose (**a**) and PMS dose (**b**). Condition: in (**a**), [catalyst] = 50–200 mg/L and [PMS] = 614 mg/L = 2 mM; in (**b**), [catalyst] = 100 mg/L, [PMS] = 1–4 mM; and [AO7] = 50 mg/L and T = 25 °C.

Figure 3a shows the effect of the catalyst dosage on AO7 degradation. Compared with the control group, the use of PMS alone without the catalyst barely had any removal effect on AO7. When the dosage of the catalyst was 50 mg/L, after 45 min of reaction, 97.0% of the AO7 was removed; when the catalyst dosage was 100 mg/L, after 15 min of reaction, 95.7% of the AO7 was removed; when the dosage was increased to 200 mg/L, AO7 removal at 10 min could reach 97.9%. The first-order rate constant (*k*) at a dosage of 200 mg/L was 38.7 × 10^−2^ min^−1^, 5 times that of 50 mg/L NRSBC800 (6.9 × 10^−2^ min^−1^). This all indicates that NRSBC800 could catalyze the oxidation reaction of PMS and that the increase in catalyst dosage would significantly improve the efficiency of AO7 removal.

Figure 3b shows the effect of PMS dosage on AO7 removal. NRSBC800 alone had a weaker adsorption effect on AO7, with the adsorption efficiency after 45 min being only 12.6%. As the mass concentration of PMS increased, AO7 oxidation improved to some extent. However, when the mass concentration of PMS increased past a certain level, the degradation efficiency became saturated, which might be a result of SO_4_^−^ consumption by the excessive PMS. When PMS concentration increased from 1 mM to 2 mM, the first-order rate constant *k* also increased from 7.6 × 10^−2^ min^−1^ to 21.4 × 10^−2^ min^−1^; as PMS dosage (4 mM) increased, the *k* value dropped to 8.9 × 10^−2^ min^−1^.

**Figure 4:**
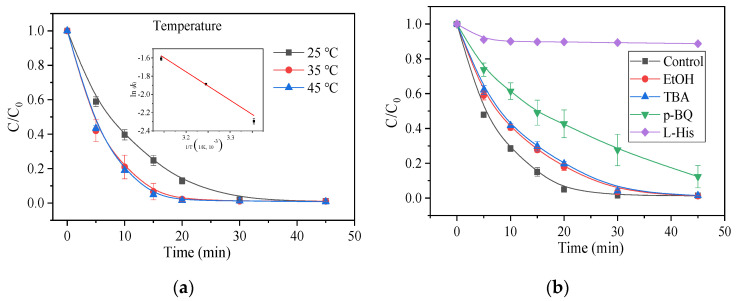
(**a**) Influences of reaction temperature; (**b**) influences of different quenchers on PMS oxidation for AO7 degradation. Condition: [AO7] = 50 mg/L, [catalyst] = 100 mg/L, [PMS] = 614 mg/L = 2 mM, T = 25 °C, [TBA] = [EtOH] = 0.5 M and [p-BQ] = [L-his] = 10 mM.

The temperature is a key contributory factor for PMS activation. In this research, the AO7 degradation was tested at different temperatures. As shown in Figure 4a, temperature can significantly influence AO7 degradation; as temperature increases, the removal rate also increases gradually. At 25 °C, AO7 removal in 30 min reached 98.1%; as the temperature continued to increase to 35 °C, AO7 removal in 20 min was as high as 97.8%; and when the temperature rose to 45 °C, AO7 removal in 15 min could reach 95.2%. The reasons behind this could be the easier activation of PMS by NRSBC800 when generating sulfate radicals and the AO7 molecule overcoming the reaction activation energy with more ease at higher temperatures. The kinetic simulation of degradation trends at different temperatures showed that the degradation fit better with first-order degradation kinetics. The reaction kinetic constant *k* at 25 °C, 35 °C and 45 °C was 10.1 × 10^−2^ min^−1^, 15.1 × 10^−2^ min^−1^ and 20.0 × 10^−2^ min^−1^, respectively; the higher temperature, the faster the degradation. The Arrhenius formula was adopted to fit *k* at different temperatures. The activation energy *E*a worked out to be 27.0 kJ/mol.

### 3.3. ROS Analysis and the Mechanism of Activation

To determine the main free radicals in the reaction system, certain amounts of p-benzoqulnone (p-BQ), L-histidine (L-His), tertiary butyl alcohol (TBA) and ethanol were added to the system as radical scavengers. Ethanol showed basically the same reaction rate as the two radicals SO_4_^•−^ and ^•^OH [61,62]; TBA only showed a quenching effect on ^•^OH [61,62]; p-BQ was a good quencher of O_2_^•−^ [44,62]; and L-His had a quenching effect on ^1^O_2_ [44,62]. As shown in Figure 4b, without free radical scavengers, the AO7 degradation rate was nearly 100% after 30 min; after introducing EtOH and TBA, the degradation rate was not significantly inhibited; p-BQ had a certain effect on the degradation rate, with the AO7 removal after 45 min being only 87.7%. Judging by the reaction rate, without free radical scavengers, the reaction rate constant *k* was 21.4 × 10^−2^ min^−1^; the reaction rate constants after adding TBA, EtOH and p-BQ were 8.0 × 10^−2^ min^−1^, 8.4 × 10^−2^ min^−1^ and 4.1 × 10^−2^ min^−1^, respectively. This all demonstrated the existence of a limited quantity of SO_4_^•−^, ^•^OH and O_2_^•−^ in the reaction system. There could be other ROS involved in the reaction of the system. Therefore, after introducing 10 mM L-His, the AO7 removal was almost completely suppressed. It could be determined, therefore, that the ROS of the reaction system were dominated by ^1^O_2_ and supplemented by SO_4_^•−^, ^•^OH and O_2_^•−^.

**Figure 5:**
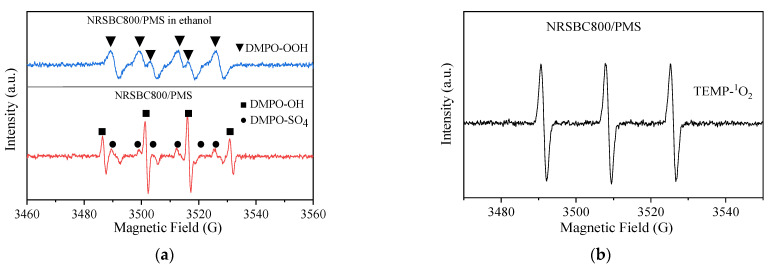
EPR spectra of DMPO-OOH, DMPO-OH and DMPO-SO_4_ (**a**) and the TEMP-^1^O_2_ adduct (**b**) formed in the NRSBC800/PMS system. (**a**) Initial reaction time and (**b**) 5 min after PMS activation. Condition: [NRSBC800] = 100 mg/L, [PMS] = 614 mg/L, T = 25 °C, [DMPO] = 20 mM and [TEMP] = 100 mM.

To further verify these results, the ROS in the catalytic system were then detected by EPR. The results are shown in Figure 5a,b. DMPO-OOH, DMPO-OH and DMPO-SO_4_ signals were detected 5 min after the NRSBC800 activation of PMS, by which the existence of SO_4_^•−^, ^•^OH and O_2_^•−^ could be deduced. More importantly, characteristic triplet signals of TEMP-^1^O_2_ were observed, indicating that there was ^1^O_2_ in the NRSBC800/PMS system. These results agreed with the free radical quenching experiment.

Generally, the form of N has an important effect on the catalytic activity of nitrogen-doped biochar. Pyridine N and pyrrole N can show high chemical activity, can transfer electrons and can activate PMS to generate free radicals, realizing the catalytic degradation of pollutants. Graphite N features higher electronegativity and a smaller covalent radius and can therefore promote electron transfer from the adjacent C, thereby producing carbon atoms with positive charge and then ^1^O_2_ with the adsorbed PMS. AO7 degradation is mainly decided by the non-radical path; therefore, graphite N might play a key role in promoting PMS activation [50].

### 3.4. The PMS-Activating Efficiency of Different Catalysts

**Figure 6:**
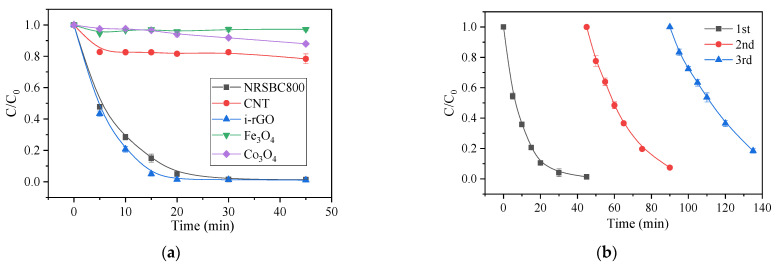
(**a**) Catalytic performances of different materials for PMS activation on AO7 removal; (**b**) degradation of the AO7 using the recycled NRSBC800. Condition: [AO7] = 50 mg/L, [catalyst] = 100 mg/L, [PMS] = 614 mg/L and T = 25 °C.

Figure 6a shows different oxidation systems for AO7 degradation. Under the same conditions, Co_3_O_4_/PMS and Fe_3_O_4_/PMS showed almost no treatment effect on AO7; the performance of the CNT/PMS system was improved to some extent, but only reached AO7 removal rates of 12% in 5 min and 22% after 45 min. The N-doped graphene i-rGO (i-rGO-N)/PMS system showed high removal performance and could completely remove AO7 after 30 min. Therefore, under selected conditions, both traditional carbon nanomaterial (CNT) and common metal catalysts (Co_3_O_4_ and Fe_3_O_4_) cannot effectively activate PMS to remove AO7. In contrast, the NRSBC800 can almost achieve the pollutant removal performance of N-doped graphene.

### 3.5. Repeated Use of Catalysts

In actual environmental applications, the stability and recyclability of the NRSBC800/PMS system for pollutant degradation seems particularly important. Repeated use of biochar was evaluated by a multi-cycle experiment using the recycled biochar again directly, and the results are shown in Figure 6b. NRSBC800 reused once and twice showed AO7 removals of 98.7% and 92.6%, respectively; after being recycled three times, AO7 removal after 45 min of reaction in the NRSBC800/PMS system dropped slightly to 81.6%. In short, the prepared catalyst NRSBC800 is of good stability and can still efficiently remove AO7 after repeated use.

### 3.6. Catalyst Applicability Test

In practice, NO_3_^−^, HCO_3_^−^ and other anions as well as humic acid (HA) and other dissolved organic matters are widespread in water. Therefore, anions and HA were added into the solution to simulate actual water and to explore their effect on the NRSBC800/PMS system’s removal of AO7. As shown in Figure S2, after adding 5 mM anions and 10 mg/L HA into the solution, the system could still remove almost all AO7.

To further evaluate the pollutant-removing effect of the NRSBC800/PMS system in actual water, water samples from two different sources (the tap water and river water at Jiaxing University, Jiaxing, China) were collected, filtered and prepared to solutions of the same concentration after adding AO7. The samples, ultrapure water and deionized water were included in the degradation experiment under the same conditions. The properties of real water samples are given in Table S3 and the experimental results are shown in Figure S3. The results demonstrated the effectiveness of the NRSBC800/PMS system in actual water samples.

### 3.7. Activation Characteristics of Different Nitrogen-Doped Straw Biochar

Figure S4 shows the AO7 degrading effects of different N-doped biochar (N-doped wheat straw biochar (NWSBC) and N-doped corn stalk biochar (NCSBC)). According to the results, the AO7 degradation by PMS activation with the catalysts from different kinds of straw showed little difference. After 45 min of reaction, the NRSBC, NWSBC and NCSBC removals of AO7 were 99.2%, 99.0% and 98.5%, respectively. However, the catalyzed reaction rate was quite different. The first-order kinetic reaction constants of the systems of NRSBC, NWSBC and NCSBC with PMS were 21.4 × 10^−2^ min^−1^, 10.1 × 10^−2^ min^−1^ and 9.2 × 10^−2^ min^−1^, respectively. Therefore, the catalysts prepared by different straw showed different performances in the PMS activation, and NRSBC was the best one.

## 4. Conclusions

In this paper, with rice straw as the raw material and urea as the nitrogen precursor, the nitrogen-doped biochar material NRSBC was synthesized by one-step pyrolysis. NRSBC can effectively activate PMS to degrade the azo-dye AO7. According to characterization of the catalysts, nitrogen doping helps increase the N content in biochar; the use of urea further improves its N content and graphite N distribution, which contributed mostly to its improved catalytic performance. According to the research, pollutant degradation follows the pseudo-first-order kinetic model; AO7 degradation rate is influenced by different experimental factors (including catalyst dosage, PMS concentration, reaction temperature, anions, HA, etc.). A quenching experiment and EPR revealed the existence of SO_4_^•−^, ^•^OH, O_2_^•−^ and ^1^O_2_ in the NRSBC/PMS oxidation system. The main ROS was ^1^O_2_, which can effectively initiate non-free radical oxidation to remove the pollutant AO7 in water. A recycling experiment verified the stability of NRSBC800. Applicability tests demonstrated NRSBC800’s effective degradation of pollutants in actual water. In general, the research findings are very useful in the development of co-doped biochar materials for PMS-based AOPs to treat water pollution.

## Data Availability

The data is available from the corresponding authors upon reasonable request.

## Supplementary Materials

The following are available online at https://www.mdpi.com/article/10.3390/nano11092288/s1, Table S1: Surface porosity of various materials, Table S2: The chemical composition of various materials, Table S3. Quality parameters of water samples, Figure S1: XPS survey spectra (a) and atomic% (b) of different materials (RSBC800, NRSBC700, NRSBC800, NRSBC900 and reused NRSBC800), Figure S2: Effect of anions and humic acid (HA) on the removal of AO7. Condition: [AO7] = 50 mg/L, [catalyst] = 100 mg/L, [PMS] = 614 mg/L, [Anions] = 5 mM, [HA] = 10 mg/L and T = 25 °C, Figure S3: Effect of actual water matrices on the AO7 degradation. Condition: [AO7] = 50 mg/L^1^, [catalyst] = 100 mg/L, [PMS] = 614 mg/L and T = 25 °C. (Abbreviations: UPW—Ultrapure Water; TW—Tap Water; DW—Deionized Water; and RW—River Water), Figure S4: Degradation of the AO7 using different kinds of straw. Condition: [AO7] = 50 mg/L, [catalyst] = 100 mg/L, [PMS] = 614 mg/L and T = 25 °C.
