# Supplementary material for: N-Doped Biochar as a New Metal-Free Activator of Peroxymonosulfate for Singlet Oxygen-Dominated Catalytic Degradation of Acid Orange 7
Source: Nanomaterials (Basel). 2021 Sep 2;11(9):2288. doi: 10.3390/nano11092288 (PMC8471211; doi:10.3390/nano11092288)
Supplement: Supplementary file 1 [file nanomaterials-11-02288-s001.zip › nanomaterials-1318207-supplementary.pdf]

nanomaterials

Supplementary Materials

N-Doped Biochar as a New Metal-Free Activator of Peroxymonosulfate for Singlet Oxygen-Dominated Catalytic Degradation of Acid Orange 7

Ruirui Han [1], Yingsen Fang [2], Ping Sun [2], Kai Xie [2,3], Zhicai Zhai [2], Hongxia Liu [1] and Hui Liu [2,*]

1 College of of Advanced Materials and Engineering, Jiaxing Nanhu University, Jiaxing 314001, China; hanruirui@zjxu.edu.cn (R.H.); liuhongxia@zjxu.edu.cn (H.L.)
2 College of Biological, Chemical Sciences and Engineering, Jiaxing University, Jiaxing 314001, China; fangyingsen@zjxu.edu.cn (Y.F.); sunping@zjxu.edu.cn (P.S.); xiekai2050956@163.com (K.X.); zhaizhicai@zjxu.edu.cn (Z.Z.)
3 College of Petroleum Engineering, Liaoning Petrochemical University, Fushun, 113001, China
* Correspondence: liuhui@zjxu.edu.cn; Tel.: +86-0573-8364-3695

Table S1. Surface porosity of various materials.

samples	SSA (m^2/g)	Pore volume (cm^3/g)	Pore size (nm)
RSBC800	428.53	0.11	3.99
NRSBC700	333.65	0.15	2.91
NRSBC800	471.12	0.13	3.30
NRSBC900	514.31	0.36	2.94

Table S2. The chemical composition of various materials.

samples	C at.%	O at.%	N at.%	N distribution %		
				Pyridine-N	Pyrrole-N	Graphene-N
RSBC800	82.98	14.47	2.55	—	—	—
NRSBC700	68.98	12.67	18.35	48.40	23.40	28.20
NRSBC800	78.71	16.42	4.87	42.04	25.28	32.68
NRSBC900	72.67	21.12	0.12	42.80	26.57	30.63
reused NRSBC800	68.19	26.92	4.89	31.85	26.39	41.76

Table S3. Qualiy parameters of water samples.

Samples	Turbidity (NTU)	UV254 (cm^{-1})	pH	Conductivity (μs/cm)
Ultrapure Water	0	0.005	6.89	2.2
Deionized Water	0	0.007	7.01	8.1
Tap Water	0.031	0.103	7.22	229
River Water	7.011	0.155	7.56	538

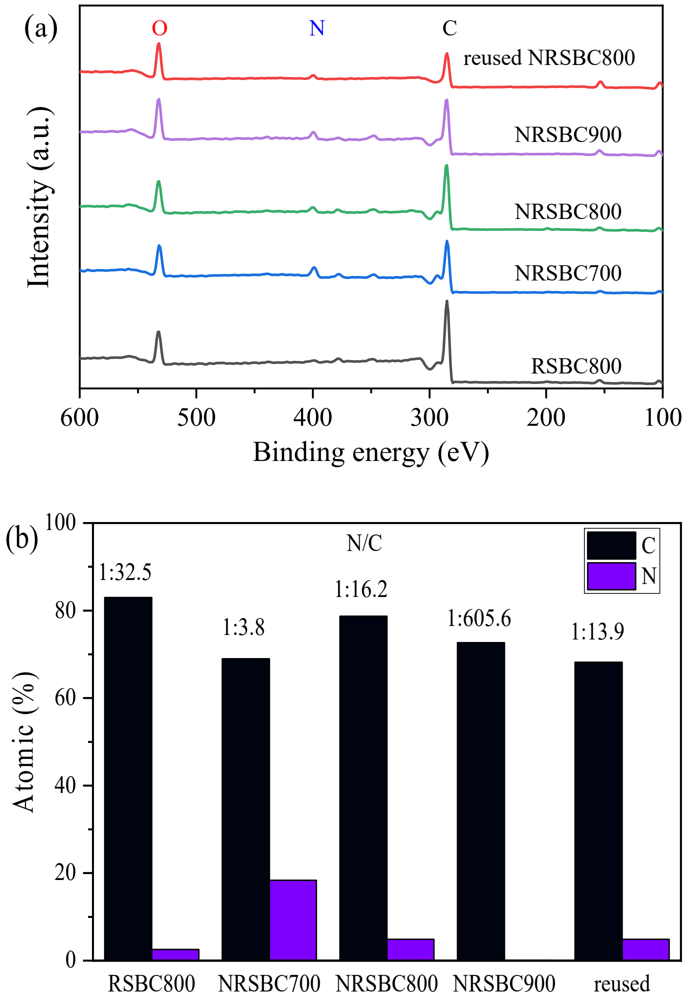

Figure S1. XPS survey spectra (**a**) and atomic% (**b**) of different materials （RSBC800，NRSBC700，NRSBC800, NRSBC900 and reused NRSBC800）.

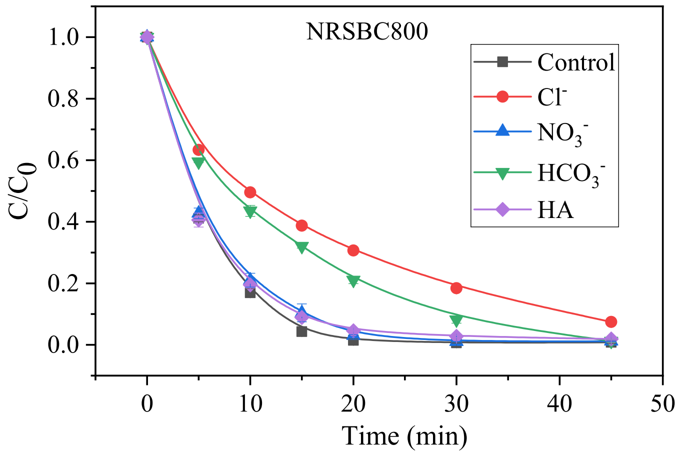

Figure S2. Effect of anions and humic acid (HA) on the removal of AO7. Condition: [AO7] = 50 mg/L, [catalyst] = 100 mg/L, [PMS] = 614 mg/L, [Anions] = 5 mM, [HA] = 10 mg/L and T = 25 °C.

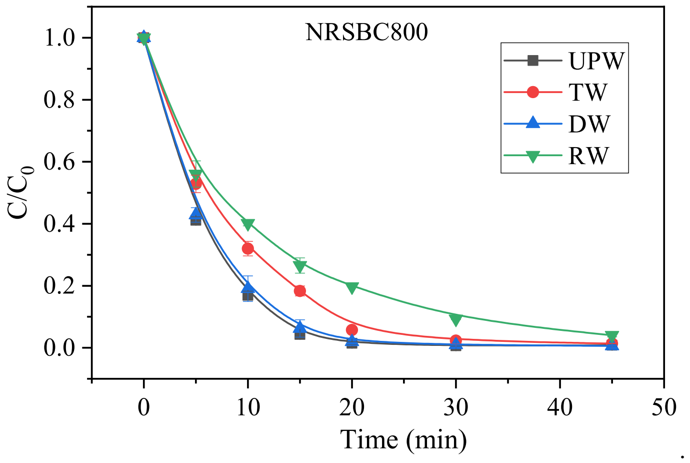

Figure S3. Effect of actual water matrices on the AO7 degradation. Condition: [AO7] = 50 mg/L, [catalyst] = 100 mg/L, [PMS] = 614 mg/L, and T = 25 °C. (Abbreviations: UPW–Ultrapure Water; TW–Tap Water; DW–Deionized Water; RW–River Water.).

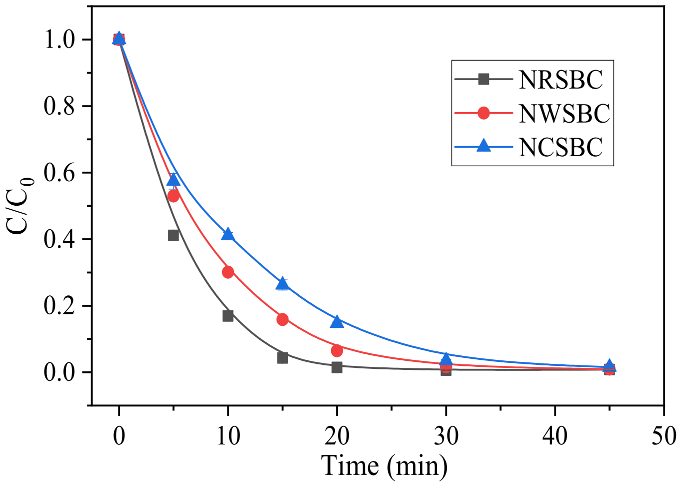

Figure S4. Degradation of the AO7 using different kinds of straw. Condition: [AO7] = 50 mg/L, [catalyst] = 100 mg/L, [PMS] = 614 mg/L, and T = 25 °C.
